# Virtual reality-based cognitive screening in psychiatry: Investigating optimal cut-offs for cognitive impairment in bipolar disorder^☆^^☆☆^

**DOI:** 10.1016/j.nsa.2025.106880

**Published:** 2025-12-25

**Authors:** Andreas Elleby Jespersen, Julie Marie Lindhardsen, Anders Lumbye, Viktoria Damgaard, Johanna Mariegaard Schandorff, Christina Mikkelsen, Maria Didriksen, Sisse Rye Ostrowski, Lars Vedel Kessing, Kamilla Woznica Miskowiak

**Affiliations:** a Neurocognition and Emotion Across Disorders of the Brain (NEAD) Centre, Psychiatric Centre Copenhagen, Frederiksberg Hospital, Copenhagen, Denmark; b Department of Psychology, University of Copenhagen, Copenhagen, Denmark; c Wide Angle Media, Copenhagen, Denmark; d Department of Clinical Immunology, Copenhagen University Hospital - Rigshospitalet, Blegdamsvej 9, Copenhagen, Denmark; e Novo Nordisk Foundation Center for Basic Metabolic Research, Faculty of Health and Medical Sciences, University of Copenhagen, Copenhagen, Denmark; f Department of Clinical Medicine, Faculty of Health and Medical Sciences, University of Copenhagen, Copenhagen, Denmark; g Copenhagen Affective Disorder Research Centre (CADIC), Psychiatric Centre Copenhagen, Frederiksberg Hospital, Copenhagen, Denmark

**Keywords:** Cognition, Cognitive impairment, Virtual reality, Cognitive screening, Bipolar disorder, Mood disorders, Ecological validity

## Abstract

Cognitive impairment is a key treatment target across neuropsychiatric disorders, including bipolar disorder (BD), but standard neuropsychological tools often fail to capture real-world cognitive challenges. Virtual reality (VR) offers a more ecologically valid alternative. This study investigated the optimal cut-off score of the novel Cognition Assessment in Virtual Reality (CAVIR) test for detecting impaired functional cognitive capacity in BD. Cut-off scores on the CAVIR were investigated in a sample of outpatients with BD (primary clinical screening BD sample; *n* = 68), using the Screen for Cognitive Impairment in Psychiatry (SCIP) as the reference for objective cognitive impairment. Receiver operating characteristic (ROC) analyses were used to identify optimal cut-off scores for detecting cognitive impairment, defined as performance ≥1 SD below the healthy controls (HC) mean (*n* = 68). Analyses were replicated in an independent mixed sample of patients with mood or schizophrenia spectrum disorders (*n* = 70) and HCs (*n* = 70), using a full neuropsychological test battery as reference. Associations between CAVIR, SCIP and interviewer-rated functioning were also explored. In the primary clinical screening BD sample, a CAVIR total score cut-off of ≤64 showed fair sensitivity (74 %) and specificity (71 %) for detecting cognitive impairment with an area under the curve (AUC) of 0.74. This cut-off score proved robust, with the optimal cut-off in the mixed replication sample of ≤66 differing by only two points. CAVIR performance was weakly to moderately associated with SCIP and functioning. The CAVIR demonstrated fair sensitivity and specificity for detecting cognitive impairment, supporting its potential as an ecologically valid screening tool for impaired functional cognitive capacity.

## Highlights

• VR-based CAVIR test proved effective in identifying impaired functional cognitive capacity among bipolar outpatients.
• CAVIR was also sensitive to impaired functional cognitive capacity in patients with mood or schizophrenia spectrum disorders.
• The study supports CAVIR as a practical, ecologically valid tool for screening impaired functional cognitive capacity in daily life.

## 1 Introduction

Cognitive impairment is a common feature and has become a key treatment target for promoting functional recovery in patients with psychiatric conditions (Miskowiak et al., 2018). In bipolar disorder (BD), impairments are evident across several cognitive domains, including memory, attention, and executive functions, and often persist even during periods of symptomatic remission (Bora et al., 2008; Bourne et al., 2013). Importantly, research has linked cognitive impairment to poorer functional outcomes, reduced quality of life and worse prognosis in BD (Tse et al., 2014). Nevertheless, cognitive impairment varies significantly between BD individuals, with some participants exhibiting global or selective cognitive impairments, while others show relatively intact cognitive functioning (Burdick et al., 2014; Kjærstad et al., 2022). The link between cognitive impairment and poorer functioning and prognosis, and the substantial inter-individual heterogeneity, underscores the importance of accurately identifying patients suffering from undetected cognitive impairment (Miskowiak et al., 2018, 2024a). In keeping with this, the International Society for Bipolar Disorders (ISBD) Targeting Cognition Task Force recommends systematic screening for cognitive impairment as an important strategy in optimizing the clinical care in BD (Miskowiak et al., 2018). Specifically, the recommendation is to conduct formal cognition assessements for all BD patients in remission using brief neuropsychological screening tools, such as the Screen for Cognitive Impairment in Psychiatry (SCIP) (Miskowiak et al., 2018; Purdon, 2005). The SCIP is currently considered gold-standard for cognitive screening in BD (Miskowiak et al., 2018) and has previously been found to be feasible in a specialized outpatient setting to identify patients with cognitive deficits and initiate cognitive strategy discussions (Miskowiak et al., 2024a). However, a key methodological challenge in the field is that performance on traditional paper-and-pencil-based cognitive tests, including the SCIP, does not fully capture patients’ real-world functioning (Jespersen et al., 2025; Miskowiak et al., 2022). Indeed, such cognitive tests are administered in a quiet, controlled environment with specific instructions, which retains little resemblance to the complex cognitive challenges that patients may face in their real-world environment (Miskowiak et al., 2022). Accordingly, traditional cognitive test batteries explain only 5–21 % of the variance in daily functioning (Van der Elst et al., 2008), limiting their effectiveness in assessing real-world cognitive performance and guiding appropriate treatment strategies (Miskowiak et al., 2022).

Virtual reality (VR) presents a novel opportunity for cognitive assessment in ecologically valid scenarios that mimic real-world situations while still maintaining high experimental control (Neguț et al., 2016; Parsons, 2015). Leveraging this potential, we developed a fully immersive, brief (≤15 min) VR cognition test, the Cognition Assessment in Virtual Reality (CAVIR), designed to evaluate everyday cognitive skills related to meal preparation in a virtual kitchen scenario (Miskowiak et al., 2022). The CAVIR has previously been shown to be a valid and sensitive tool for assessing cognitive impairment in individuals with psychiatric disorders, including BD (Jespersen et al., 2025; Miskowiak et al., 2022). Further, CAVIR performance was linked to performance-based activity of daily living (ADL) ability, whereas standard neuropsychological test performance was not, suggesting a potential of CAVIR to bridge between (abstract) neuropsychological performance and (real-world) functional capacity to better capture patients’ cognitive skills in real-world situations (Jespersen et al., 2025). Together, these previous findings suggest that CAVIR may tap into its own distinct but related construct, here referred to as functional cognitive capacity (FCC), defined as the capacity to apply cognitive functions in challenging daily life activities (Jespersen et al., 2025). Building on these promising findings, a key next step is to assess the utility of CAVIR as a cognitive screening tool in a clinical outpatient setting.

To address this, we conducted post-hoc analyses of data from a cognitive screening programme at the Copenhagen Affective Disorder Clinic (AAL), where outpatients with newly diagnosed BD completed both the CAVIR and the SCIP. The study aimed to determine (I) the CAVIR cut-off score with optimal sensitivity and specificity for detecting cognitive impairment according to the gold-standard cognitive screening instrument, SCIP, using Receiver Operating Curves (ROC) and (II) the association between performance on the CAVIR and SCIP and interview-rated functioning. Finally, the study aimed to (III) test the reliability of the identified CAVIR cut-offs in the clinical screening sample through a ROC analysis in a separate ‘replication sample’ with a mix of patients with mood or schizophrenia spectrum disorders who completed a comprehensive neuropsychological assessment. This approach was not intended to position CAVIR as a full neuropsychological assessment, but rather to examine the robustness of the identified CAVIR cut-off scores to screen for impaired FCC across independent samples.

## 2 Methods

### 2.1 Participants and recruitment

#### 2.1.1 Clinical screening bipolar disorder sample

The sample comprised outpatients with BD recruited through a cognitive screening program which targets newly diagnosed patients (<2 years since diagnosis or < 30 years of age) that have received treatment for 15–20 months at the Copenhagen Affective Disorder Clinic (AAL), Mental Health Services, Capital Region of Denmark (Miskowiak et al., 2024a). The rationale for conducting the cognitive screening as this illness stage is to target persistent cognitive impairment that do not result from acute mood symptoms. Further details on this cognitive screening program are provided in our previous publication (Miskowiak et al., 2024a). Participants were eligible if they had an ICD-10 diagnosis of BD (type I or II) which was verified by consultant psychiatrists with specialist training at AAL and confirmed through psychiatric records, were 18–70 years of age and fluent in Danish. No formal criteria for symptom stability were applied. However, in accordance with the cognitive screening program, participants were only included after having received specialized outpatient treatment for 15–20 months at which time most patients were expected to be in partial or full remission. No further in- or exclusion criteria were applied to ensure that the sample was representative of patients with BD receiving routine outpatient care in a Danish clinical setting. Data from healthy control participants (HCs) matched on age, education and sex was extracted from the databases of our prior study on normative values for the SCIP (Ott et al., 2021). In this study, HCs were excluded based on self-reported personal or first-degree family history of psychiatric illness, although this was not confirmed using a structured diagnostic interview.

The ethics committee in the Capital Region of Denmark deemed that formal ethics approval was not required as the assessments were offered as part of patients’ standard care and involved no biomedical devices or invasive procedures (P-2022–356).

#### 2.1.2 Replication sample of patients with mood or schizophrenia spectrum disorders

The replication sample comprised a mixed sample of patients with an ICD-10 diagnosis of either BD, unipolar disorder or a schizophrenia spectrum disorder and HCs. Data was collected from the baseline assessments of two ongoing clinical trials with shared inclusion criteria and assessment batteries (for full details, see the respective study protocols (Jespersen et al., 2024; Miskowiak et al., 2024b)). Notably, the mixed replication sample is identical to the cohort included in our previous study that aimed to validate the CAVIR test (Jespersen et al., 2025). However, the present study reports distinct and novel analyses focusing specifically on identifying optimal cut-off scores for cognitive impairment for clinical application. In brief, all participants were 18–55 years of age and fluent in Danish. Patients were recruited from outpatient clinics in the Capital Region of Denmark and their diagnoses were verified with the Schedules for Clinical Assessment in Neuropsychiatry (SCAN) (Wing et al., 1990). Patients with BD or UD were in full or partial remission upon inclusion (scores ≤14 on the Hamilton Depression Rating Scale 17 items (HDRS-17) (Hamilton, 1960) and Young Mania Rating Scale (YMRS) (Young et al., 1978)) and patients with SSD were assessed to be relatively symptom stable by their referring clinician. In addition, patients were required to present with clinically relevant subjective and/or objective cognitive impairment (see study protocols for details (Jespersen et al., 2024; Miskowiak et al., 2024b)). The HC participants in the replication sample were recruited through website advertisements or from blood banks in the Capital Region of Denmark and were free of any personal history of psychiatric illness (confirmed with SCAN). General exclusion criteria were current alcohol/substance use, neurological disorder, severe somatic illness, dyslexia, a daily use of benzodiazepines >22,5 mg oxazepam, and electroconvulsive therapy (ECT) during the last three months. Both trials were approved by the local ethics committee in the Capital Region of Denmark (protocol numbers: H-22004153 and H-22028111).

For all studies, written informed consent was obtained from all participants prior to study participation.

### 2.2 Procedure

#### 2.2.1 Clinical screening sample of outpatients with bipolar disorder

The cognitive screening program was developed in accordance with recommendations put forth by the ISBD Targeting Cognition Task Force (Miskowiak et al., 2018) and involved offering cognitive screenings with feedback to newly diagnosed outpatients with BD enrolled in treatment at the AAL (Miskowiak et al., 2024a). As part of this program, patients were screened 15–20 months into their two-year treatment schedule at a 1.5 h session comprising the CAVIR, SCIP, and a rating of functional capacity (further details below). Mood symptoms were assessed with the HDRS-17 and YRMS. At the end of the session, all patients received oral and written feedback focusing on strategies for supporting their cognitive reserve and compensating for potential cognitive impairment (Miskowiak et al., 2018, 2024a).

#### 2.2.2 Replication sample of patients with mood or schizophrenia spectrum disorders

Assessments were conducted in 2 h sessions as part of the baseline assessement in the aforementioned clinical trials and comprised the CAVIR and a traditional neuropsychological test battery (details below). Participants also underwent ratings of functional capacity and mood symptoms with the HDRS-17 and YRMS. Participants in the replication sample did not complete the SCIP as part of this assessment.

### 2.3 Materials

#### 2.3.1 Cognition Assessment in Virtual Reality (CAVIR)

The CAVIR is a fully immersive 360° VR cognition test assessing daily life cognitive skills in a virtual kitchen environment (Jespersen et al., 2025; Miskowiak et al., 2022). The test is self-administered (≥15 min) using a standalone Meta-Quest 128 GB headset. A pre-recorded voice guides the participant through five sub-tasks assessing different cognitive functions in the context of meal preparation (Jespersen et al., 2025). Task 1 measures verbal memory and involves memorization of an ingredient list. Task 2 measures executive functions by having participant plan different subtasks related to meal preparation. Task 3 measures processing speed and involves adding correct ingredients to a pot as quickly as possible in a set amount of time using a symbol-ingredient key. Task 4 measures working memory and requires the participant to memorize where kitchen utensils and flatware are located. Task 5 assesses sustained attention and involves ensuring that the food does not get burned by paying attention to visual and auditive cues while ignoring irrelevant stimuli. CAVIR was developed in two parallel versions, which have previously demonstrated an equal difficulty level (Jespersen et al., 2025). Patients in the clinical screening sample all received the same CAVIR version, while patients in the replication sample received one of the two parallel equivalent versions in a randomized design. For more details on the CAVIR, see (Jespersen et al., 2025).

#### 2.3.2 Standard cognitive assessments

Cognitive screening of outpatients with BD was conducted with the SCIP (Danish version) in line with the recommendations by the ISBD Targeting Cognition Task Force (Miskowiak et al., 2018). The SCIP is a brief (<20 min) paper-and-pencil tool that has been validated for the detection of cognitive impairment in BD, demonstrating high decision validity, internal consistency, test-retest reliability, and concurrent validity (Purdon, 2005; Jensen et al., 2015). SCIP comprises five subtasks measuring verbal learning and memory (VLT-I), verbal working memory (WMT), verbal fluency (VFT), delayed memory (VLT-D), and psychomotor speed (PST) (Purdon, 2005).

The neuropsychological test battery administered in the mixed replication sample, comprised the following traditional neuropsychological tests: The CANTAB (Cambridge Cognition Ltd.) One Touch Stocking of Cambridge (OTS), Spatial Working Memory test (SWM) and Rapid Visual Information Processing (RVP). The Rey Auditory Verbal Learning Test (RAVLT) (Schmidt, 1996), Letter-Number-Sequencing from WAIS-III (Wechsler, 1997), RBANS Coding and Digit span (Randolph et al., 1998), verbal fluency with letters ‘d’ and ‘s’ (Borkowski et al., 1967) and Trail Making A and B (Army Individual Test Battery, 1944). The Danish Adult Reading Test (DART), was administered to assess premorbid verbal IQ (Crawford et al., 1987). For more details, see p. 3 in **Supplementary Materials**.

#### 2.3.3 Assessment of functioning

Functional capacity was assessed in both samples using the interviewer-rated Functioning Assessment Short Test (FAST) (Rosa et al., 2007), a sensitive measure of functional impairment in BD recommended by the ISBD Targeting Cognition Task Force (Miskowiak et al., 2017).

### 2.4 Statistical analyses

Total scores for the CAVIR and the SCIP, were determined by totaling the five subtest-raw scores of the respective instrument. For tests with lower scores indicating better performance, the scores were inversed to ensure that all scales had the same direction. Raw scores on the SCIP were *z*-transformed based on the mean and SD of the HCs. Raw scores on the standard neuropsychological tests in the replication sample were also z-transformed based on the mean and SD of the HCs and five cognitive domains were calculated by averaging the *z*-transformed scores within each subtask. A global neuropsychological score was then calculated by averaging the five domains. See p. 3–4 in **Supplementary Materials** for further details.

Continuous data were assessed for normality using Shapiro-Wilk tests, and non-parametric tests were conducted whenever the assumption of normality was violated. Groups were compared on demographic and clinical variables using independent sample t-tests or Mann-Whitney U tests for normally and non-normally distributed data. A Chi-square (χ^2^) test was used to examine group differences in sex distribution (male/female as assigned at birth).

To evaluate the utility of CAVIR for cognitive screening, Receiver Operating Characteristic (ROC) analyses were conducted. This method assesses the ability of the instrument to distinguish between patients classified as cognitively impaired or intact based on established cognitive measures. Each potential cut-off score is associated with sensitivity and specificity values, reflecting the proportion of true positives and true negatives for objective cognitive impairment (Metz, 1978). The analyses aimed to identify the CAVIR total score cut-off that optimally balances sensitivity and specificity. Objective global cognitive impairment was defined as a total score ≥1 SD below the HC mean on the SCIP for the clinical screening BD sample (aim I) since this is the cutoff typically recommended in clinical settings (Miskowiak et al., 2018, 2024a). In the replication sample (aim III), cognitive impairment was defined as a score of ≥1 SD below the HC mean on the global neuropsychological composite score. Finally, correlation analyses (Pearson's *r* or Spearmans ρ (*r*_*s*_)) was applied in the clinical screening sample to examine how performance on the CAVIR (total score) was associated with total SCIP performance and FAST scores, respectively (aim II). Post-hoc multiple regression analyses were performed in cases of significant correlations, to investigate if CAVIR performance was still a significant predictor of SCIP performance and interviewer-rated functioning when controlling for age and depressive symptoms.

All statistical analyses were carried out using IBM SPSS statistics V.29 with the threshold for statistical significance were set at p ≤ 0.05 (two-tailed).

## 3 Results

### 3.1 Demographics and clinical variables

Table 1 outlines demographic and clinical data. We included *n* = 68 patients in the clinical screening sample. The patients were comparable to the HCs (*n* = 68) on age and sex (*p*_*s*_ ≥ 0.96) but displayed more symptoms of (hypo)mania (*U* = 1197.0, *p* < 0.001) and depression (*U* = 651.0, *p* < 0.001), as expected. Nevertheless, the mean HDRS-17 and YMRS total score in patient group was <14 indicating that patients were generally in full or partial remission (Table 1).

**Table 1:** Demographic and clinical characteristics in the clinical and replication samples.

	Clinical screening sample			Replication sample		
	Patients (*n* = 68)	HCs (*n* = 68)	*p*-values	Patients (*n* = 70)	HCs (*n* = 70)	*p*-values
*Demographics*						

Sex (female/male) (%)	62/38	62/38	1.00	50/20	43/27	0.21
Age in years, mean (SD)	31.9 (10)	31.9 (10)	0.96	33.4 (10.9)	30.3 (8.9)	0.19
Educational years, mean (SD)	14.6 (2.5)	15.4 (2.5)	0.04∗	14.4 (2.5)	15.6 (2.2)	<0.01∗∗
Est. premorbid verbal IQ	–	–	–	111.8 (5.7)	111.4 (5.2)	0.637

*Clinical characteristics*						

Bipolar type 1, no (%)	14 (20.6)	–	–	10 (14.3)	–	–
Bipolar type 2, no (%)	50 (73.5)	–	–	26 (37.1)	–	–
Bipolar type unknown, no (%)	4 (5.9)	–	–	0 (0.0)	–	–
Unipolar disorder, no (%)	–	–	–	16 (22.9)	–	–
Schizophrenia, no (%)	–	–	–	7 (10.0)	–	–
Schizotypal disorder, no (%)	–	–	–	9 (12.9)	–	–
Unspecified non-organic psychosis, no (%)	–	–	–	2 (2.9)	–	–
HDRS-17, mean (SD)	8.6 (6.7)	1.2 (1.4)	<0.001∗∗	4.4 (3.8)	0.8 (1.1)	<0.001∗∗
YMRS, mean (SD)	3.1 (3.5)	0.6 (1.0)	<0.001∗∗	1.0 (1.8)	0.4 (1.0)	0.09

*Cognition*						

CAVIR total, mean (SD)	63.8 (14.4)	–	–	63.8 (16.9)	78.0 (11.3)	<0.001∗∗
CAVIR verbal learning and memory, mean (SD)	12.1 (2.5)	–	–	12.0 (2.2)	13.2 (1.4)	<0.001∗∗
CAVIR executive functioning, mean (SD)	5.7 (3)	–	–	6.1 (2.6)	6.1 (2.6)	0.92
CAVIR processing speed, mean (SD)	34.3 (7.1)	–	–	34.01 (9.0)	39.9 (7.5)	<0.001∗∗
CAVIR working memory, mean (SD)^a^	8.3 (3.4)	–	–	8.8 (5.3)	6.6 (2.2)	<0.001∗∗
CAVIR sustained attention, mean (SD)	20 (6.8)	–	–	20.6 (6.0)	25.3 (3.2)	<0.001∗∗
SCIP total, mean (SD)	78 (11)	79.7 (7.3)	0.30	–	–	
Neuropsychological global composite z-score	–	–	–	−0.7 (0.7)	0.0 (0.5)	<0.001∗∗

*Daily functioning*						

FAST total, mean (SD)	19 (12.2)	–	–	23.5 (11.3)	1.5 (2.5)	<0.001∗∗

**Notes and abbreviations**: HDRS-17 = Hamilton Depression Rating Scale 17-items version. YMRS=Young Mania Rating Scale. CAVIR = Cognition Assessment in Virtual Reality. SCIP=Screen for Cognitive Impairment in Psychiatry. FAST=Functioning Assessment Short Test. M = mean. SD = standard deviation. ∗ = p < 0.05 (two-tailed), ∗∗ = p < 0.001 (two-tailed). ^a^For the CAVIR working memory task, lower score indicate better performance.

The mixed replication sample comprised *n* = 70 patients with an ICD-10 diagnosis of either BD (*n* = 36), unipolar disorder (UD; *n* = 16) or a schizophrenia spectrum disorder (SSD; *n* = 18) and *n* = 70 HCs. Patients in the replication sample were comparable to HCs on age, sex, premorbid verbal IQ, and YMRS scores (*p*_*s*_ ≥ 0.09), but had fewer years of education (*t* (138) = 3.06, *p* < 0.01) and displayed more subsyndromal symptoms of depression, as indicated by higher HDRS-17 scores (*U* = 719.0, *p* < 0.001; Table 1).

A comparison of patient groups in the cognitive screening and replication samples is provided in Table S1 in the **Supplementary Materials**.

### 3.2 Optimal CAVIR cut-off score for cognitive impairment

**Fig. 1:**
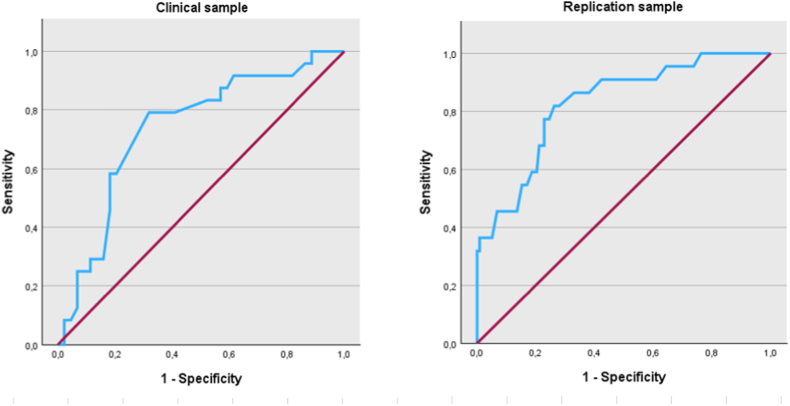
ROC curves for CAVIR total scores in the clinical screening sample (left) and the replication sample (right) applying a threshold for global cognitive impairment of ≥1 SD below the HC mean on the SCIP total score (clinical screening sample) and global neuropsychological test performance (replication sample).

Based on the ROC analysis in the clinical screening sample (see Fig. 1 and Table 2), we propose a CAVIR cut-off score for cognitive impairment of ≤64. This cutoff provided fair sensitivity (75.0 %) and specificity (70.5 %) for cognitive impairment according to the SCIP, with an area under the curve (AUC) of 0.74, indicating a classification accuracy of 74 % when randomly selecting a truly impaired (SCIP total ≥1 SD below the HC mean) and a non-impaired participant (see Fig. 1 and Table 2).

**Table 2:** Cut-off scores and associated values of sensitivity and specificity and AUC for the CAVIR total score in the clinical screening sample (light grey columns) and the replication sample (dark grey columns).

Cut-off score		Sensitivity		Specificity		AUC^a^		95 % Cl	
Clinical Sample	Replication sample	Clinical Sample	Replication sample	Clinical Sample	Replication sample	Clinical Sample	Replication sample	Clinical Sample	Replication sample
<62	<65	58.3	68.2	79.5	78.8	0.74	0.83	[0.612- 0.862]	[0.735- 0.919]
<63	<66	66.7	68.2	75.0	77.1				
**< 65**	**< 67**	**75.0**	**77.3**	**70.5**	**77.1**				
<66	<68	79.2	77.3	68.2	75.4				
<67	<69	79.2	81.8	63.6	73.7				

**Notes and abbreviations**: Results of the ROC analysis in the clinical and replication samples are reported in light grey and dark grey columns, respectively. AUC = Area under the curve. 95 % Cl = 95 % Confidence Interval.

a AUC: 0.90–1.00 = Excellent, 0.80–0.90 = Good, 0.70–0.80 = Fair,0.60–0.70 = Poor, 0.50–0.60 = Fail.

In the replication sample, a cut-off score of ≤66 on the CAVIR yielded optimal sensitivity and specificity of 77.3 % and 77.1 %, respectively, for cognitive impairment according to neuropsychological tests (AUC = 0.83; Fig. 1 and Table 2).

### 3.3 Associations between the CAVIR and SCIP

CAVIR total scores correlated weakly to moderately with SCIP total scores (*r* (66) = 0.38, *p* < 0.01) in the clinical screening sample of BD patients. Adjusting for age and depressive symptoms revealed a significant model (*R*^*2*^ = 0.19, *F* (3,64) = 4.9, *p* = 0.004), with total CAVIR score being the only significant predictor of SCIP performance (*β* = 0.37, *p* < 0.01); for remaining predictors *p*_s_ ≥ 0.10).

### 3.4 Associations between the CAVIR and FAST

Lower CAVIR total scores were also weakly associated with more functional disability as indexed by higher FAST total scores (*r*_*s*_ (64) = −0.29, *p* < 0.05) in the clinical screening sample of BD patients, but this association did not survive in a model adjusting for age and depressive symptoms (*p* = 0.20).

## 4 Discussion

This study investigated for the first time the clinical utility of the Cognition Assessment in Virtual Reality (CAVIR) as an ecologically valid cognitive skill assessment in outpatients with BD. ROC analysis in the sample of BD outpatients in the clinic yielded an optimal cut-off score on the CAVIR for cognitive impairment of ≤64, which was associated with fair sensitivity (75 %) and specificity (71 %). A similar threshold for cognitive impairment of ≤66 on the CAVIR was identified in the replication sample of patients with mood or schizophrenia spectrum disorders. CAVIR performance correlated weakly-to-moderately with performance on the SCIP, and with interviewer-rated functioning, although the association with functioning did not survive adjustment for age and depressive symptoms.

Compared to prior validation studies of the gold-standard SCIP, the ROC analysis in our primary BD screening sample revealed slightly lower (i.e., fair) decision validity of the CAVIR at the optimal cut-off. While SCIP cut-offs have consistently shown high sensitivity and specificity when cognitive impairment is defined as ≥1 SD below the HC mean (Jensen et al., 2015; Ott et al., 2016; Rojo et al., 2010), a Danish study in BD patients reported even higher sensitivity and specificity of 81 % and 76 %, respectively (Jensen et al., 2015). The somewhat lower - but still acceptable - decision validity of the CAVIR can reflect that it measures a related but distinct construct, namely functional cognitive capacity (FCC), defined as the capacity to apply cognitive functions in challenging daily life activities (Jespersen et al., 2025). In this respect, the lower specificity of the CAVIR may indicate that it is sensitive to daily life cognitive difficulties, which traditional neuropsychological assessment tools like the SCIP might not capture. Moreover, differences in the cognitive domains assessed may also account for these discrepancies. The CAVIR, for example, evaluates attention and spatial working memory - domains not covered by the SCIP (Jespersen et al., 2025; Jensen et al., 2015). This may explain why the sensitivity and specificity of the CAVIR were higher in the replication sample using a broader neuropsychological battery (77.3 % and 77.1 %, respectively), which includes domains overlapping with the CAVIR. Similarly, the somewhat lower convergent validity observed here relative to previous validation studies (Jespersen et al., 2025; Miskowiak et al., 2022) can likely be attributed to differences in the comparison measures. Earlier studies employed broader neuropsychological batteries, enhancing construct overlap with the CAVIR (Jespersen et al., 2025; Miskowiak et al., 2022). The SCIP, while a useful screening tool, provides only a limited assessment of cognitive domains (Miskowiak et al., 2018; Purdon, 2005). The lack of association between the CAVIR and the FAST also diverges from prior findings, which showed weak to moderate correlations (Jespersen et al., 2025; Miskowiak et al., 2022). However, this aligns with broader literature suggesting weak associations between objective cognitive performance and observer- or self-rated functioning, potentially due to confounding by residual mood symptoms (Jensen et al., 2015; Ott et al., 2016) and insight variability (Petersen et al., 2019). Moreover, FAST includes broad functional outcomes like interpersonal functioning that may be influenced by factors other than cognition. These findings underscore the need for future validation studies to also include objective performance-based functional measures, such as the Assessment of Motor and Process Skills (AMPS) (Fisher et al., 2012). Notably, our recent study demonstrated a moderate association between CAVIR and AMPS scores (ADL ability related to household tasks), supporting its ecological validity (Jespersen et al., 2025). Further, CAVIR differentiated between patients with and without the ability to undertake regular employment (Jespersen et al., 2025). These findings suggest a potential of CAVIR to better capture patients' cognitive skills in real-world situations (i.e., FCC). In this respect, CAVIR appears to measure what is often referred to ‘functional capacity’ in the schizophrenia literature, which is proposed to mediate the relationship between cognition and functioning (Bowie et al., 2010). A seemingly related construct ‘functional cognition’ has also been described in the field of occupational therapy, referring to how cognitive abilities are applied during everyday activities (Giles et al., 2024). Taken together, the current findings further support these interpretations that CAVIR might capture an its own construct (FCC) that bridges neurocognitive performance and functional outcomes, rather than serving as a direct proxy for either construct alone.

From a clinical perspective, CAVIR may offer healthcare professionals valuable patient-centered insights into their FCC and hence whether they have difficulties managing cognitively demanding real-world situations. As a performance-based ecologically valid tool, CAVIR thus represents a promising supplement to traditional self-report questionnaires and paper-and-pencil neuropsychological assessments, such as the SCIP. Given its ecological validity, the profile of impairment on the CAVIR may also guide personalized care by helping patients recognize their cognitive challenges and develop compensatory strategies in everyday life, in line with recommendations from the ISBD Targeting Cognition Task Force (Miskowiak et al., 2018, 2022). Importantly, a recent study reported low (∼50 %) response rates among BD outpatients to invitations for cognitive screening (Miskowiak et al., 2024a). In this context, incorporating CAVIR - delivered through immersive, engaging VR technology - may help increase patient motivation and participation, though further research is needed to confirm this. A further advantage of the CAVIR is its potential cost-effectiveness for assessing FCC. Gold-standard performance-based functional measures, such as the AMPS, are resource intensive and often not feasible, requiring access to real-world environments and the involvement of certified occupational therapists or trained examiners. While established cognitive screening tools such as the SCIP are short (15 min) and feasible, they still require administration and scoring by trained clinician but fail to capture real world functioning. In comparison, CAVIR is self-administered, features fully automated scoring and uses relative affordable VR equipment (∼300 €) (Miskowiak et al., 2022). This makes CAVIR a practical, brief, and scalable tool that can be easily implemented in clinical settings with minimal training or input from clinicians to gain insight into patients’ FCC. However, it remains important to determine whether CAVIR is equally suitable for populations with limited familiarity with digital or virtual reality platforms when used in routine clinical assessment. While our previous work has demonstrated that the CAVIR is feasible and sensitive to FCC impairment in patients up to at least 55 years of age (Jespersen et al., 2025; Miskowiak et al., 2022), we are currently investigating whether this extends to older patient populations.

A key strength of the study lies in its real-world clinical context, which aligns with the primary aim of evaluating the utility of CAVIR in routine care. Unlike prior validation studies conducted in controlled settings with more homogeneous samples (Jespersen et al., 2025; Miskowiak et al., 2022), this study assessed CAVIR's performance in a diverse, ecologically valid environment. Additionally, the replication of the ROC analysis in a research-based sample using a well-established cognitive battery further reinforces the robustness of the findings. Notably, the clinical screening sample comprised only patients with bipolar disorder, whereas the replication sample was diagnostically heterogeneous and drawn from baseline assessment of cognitive intervention trials, which limit direct comparability between samples. However, the patient groups did not differ in age, educational attainment, or cognitive performance, although patients in the clinical screening sample exhibited higher levels of subsyndromal manic and depressive symptoms (Table S1). Other limitations of the study include the relatively small sample size which may affect generalizability of the findings (De Prisco et al., 2024). The findings should also be interpreted with caution as the analyses were post-hoc. Further, the heterogeneity of the screening sample and the naturalistic assessment setting may have introduced greater variability than typically seen in controlled trials, potentially contributing to discrepancies in findings. In addition, the inclusion rate in the cognitive screening programme was approximately 50 % (Miskowiak et al., 2024a), which may introduce selection bias in the current clinical screening sample, as not all eligible outpatients accepted or were able to complete screening. In addition, the predominance of younger, recently diagnosed participants may further limit generalizability (Miskowiak et al., 2024a), especially to older adults who may be less familiar with VR technology (Miskowiak et al., 2022). Further, the use of a single cut-off score does not account for age-related cognitive decline, requiring caution when interpreting low scores in older individuals (Miskowiak et al., 2022; Jensen et al., 2015). Lastly, due to limited overlap between the cognitive domains assessed by CAVIR and those covered by the SCIP, subtest-specific cut-offs could not be established. This restricts the capacity to provide personalized feedback and tailor interventions, which is particularly important given the heterogeneity of cognitive deficits in BD (Burdick et al., 2014; Russo et al., 2017; Sole et al., 2016). Future research should explore optimal subtest-specific cut-offs to enhance clinical applicability.

In conclusion, this study determined the optimal cutoff on the CAVIR to screen for impaired FCC in patients’ daily lives in two separate samples, which revealed high consistency across these patient samples (cut-off scores of ≤ 64 and 66, respectively). The findings support the validity of the CAVIR as a screening tool for impaired FCC, offering a feasible and real life-like alternative to traditional cognitive tests. By assessing real-world cognitive skills, the CAVIR may help clinicians better understand the challenges patients face in daily life and tailor compensatory or cognitive training strategies accordingly. As such, the CAVIR could help bridge the gap between cognitive screening and functional outcomes, ultimately supporting improved everyday functioning and quality of life. Future research should continue to validate the CAVIR across broader clinical populations and explore its associations with performance-based functioning in routine settings.

## Data availability statement

The data that support the findings of this study are available from the corresponding author upon reasonable request.

## Ethics approval and consent to participate

For the cognitive screening programme, the ethics committee in the Mental Health Services Capital Region of Denmark stated that formal ethics approval was not required as the study involved no biomedical devices or invasive procedures and thus posed minimal risks for participants. The study was approved by the Danish Data Protection Agency at the Capital Region of Denmark (P-2022–356). The virtual reality-based cognitive remediation trial and hypoxia cognition training trial was both approved by the Committee on Health Research Ethics in the Capital Region of Denmark (protocol number: H-22004153 and H-22028111, respectively) and by the Danish Data Protection Agency at the Capital Region of Denmark (protocol number: P-2022-411 and P-2022-354, respectively). All studies were conducted in compliance with existing laws on data protection. Participants were given written and verbal information about the studies to make an informed decision about their participation. Written informed consent was obtained from all participants. The studies were conducted in accordance with the recommendations of the Declaration of Helsinki.

## CRediT authorship contribution statement

**Andreas Elleby Jespersen:** Data curation, Formal analysis, Investigation, Project administration, Software, Visualisation, Writing – original draft, Writing – review & editing. **Julie Marie Lindhardsen:** Data curation, Formal analysis, Investigation, Visualisation, Writing – original draft, Writing – review & editing. **Anders Lumbye:** Software, Writing – review & editing. **Viktoria Damgaard:** Data curation, Investigation, Project administration, Writing – review & editing. **Johanna Mariegaard Schandorff:** Data curation, Investigation, Project administration, Writing – review & editing. **Christina Mikkelsen:** Resources, Writing – review & editing. **Maria Didriksen:** Resources, Writing – review & editing. **Sisse Rye Ostrowski:** Resources, Writing – review & editing. **Lars Vedel Kessing:** Resources, Writing – review & editing. **Kamilla Woznica Miskowiak:** Conceptualisation, Data curation, formal analysis, funding acquisition, Investigation, Methodology, Project administration, resources, Software, Supervision, Validation, Visualisation, Writing – original draft, Writing – review & editing.

## Role of the funding source

The data was collected through studies supported by the 10.13039/501100007437Tryg Foundation (grant no. 150128), the Axel Muusfeldts Foundation (grant no. 2022-0097), the Jascha Foundation (grant no. 2022-0134), the Ivan Nielsen foundation (grant no. not available), the Familien Hede Nielsen Foundation (grant no. 2023-1741) and a European Research Council (ERC) Consolidator Grant (grant no. 101043416). The funding bodies had no role in the data collection, analysis, or interpretation of data, in the writing of the article, or in the decision to submit the article for publication.

## Declaration of interest statement

Kamilla Miskowiak has received honoraria from Lundbeck, Angelini, Gedeon Richter, and Janssen-Cilag in the past three years. Andreas Elleby Jespersen has received honoraria from Lundbeck in the past year. Lars Vedel Kessing has received honoraria from Lundbeck and Teva in the past three years. The remaining authors report no financial relationships with commercial interests.
