# Supplementary material for: Virtual reality-based cognitive screening in psychiatry: Investigating optimal cut-offs for cognitive impairment in bipolar disorder
Source: Neurosci Appl. 2025 Dec 25;5:106880. doi: 10.1016/j.nsa.2025.106880 (PMC12885629; doi:10.1016/j.nsa.2025.106880)
Supplement: Multimedia component 1 [file mmc1.docx]

**Supplementary methods**

*The CAVIR test – technical setup*

The CAVIR test is administered on a standalone head-mounted Meta Quest 128 GB portable headset (costing around $300) running on a fast-switch LCD display with a resolution 1832 x 1920 pixels per eye at 90hz refresh rate. The headset uses a Qualcomm Snapdragon XR2 chipset as the main CPU and GPU processing unit. To navigate the environment, the participant uses a hand-held controller. The headset supports different interpupillary distances, which can be set at 58, 63, and 68 mm depending on the user. The CAVIR test was developed by our research group at the Neurocognition and Emotion in Affective Disorders (NEAD) group at Psychiatric Center Copenhagen, Frederiksberg, and the Department of Psychology, University of Copenhagen in collaboration with virtual reality developer, Anders Lumbye, at Wide Angle media. From Miskowiak et al. 2022 [1] and Jespersen et al. 2025 [2].

*The Cognition Assessment in Virtual Reality (CAVIR)*

Before putting on the virtual reality headset, participants are instructed that the CAVIR test involves five tasks in a simulated kitchen and that they will receive instructions in the headset before the tasks begin. They are told to imagine that they have invited guests over for dinner and must complete several tasks in time for their guests’ arrival. They are instructed to stand on a designated spot on the floor and are shown the Meta Quest headset. They are also shown how to use the hand-held controller with the index finger of their dominant hand. They are told that they can ask questions before starting the test but not during the test, which will take around 15 minutes in total. The researcher helps the participant put on the headset, after which the participant is given a few minutes to orient themselves in the virtual kitchen and adjust to the environment. When they are ready, the participant can start the tasks by clicking on “Begin”.

- Verbal learning is assessed in task 1 in which participants are shown a list of ingredients for 50 seconds and instructed to memorize these and then find them in the kitchen.
- Executive functions are assessed in task 2 in which participants are required to plan and select the order in which to perform different sub-tasks involved in cooking a meal to finish before their guests’ arrival.
- Processing speed is assessed in task 3 in which participants place as many correct ingredients as possible in a pot within 90 seconds based on a key of symbols matching every ingredient.
- Working memory is assessed in task 4 in which participants observe and memorize the location of cutlery and flatware in the kitchen cupboards and drawers. They must then find the correct cutlery and flatware by only opening the correct cupboards and drawers.
- Sustained attention is measured in task 5 in which participants are required to repeatedly check the food in the oven or regulate the oven temperature in response to a specific combination of visual and auditive cues while ignoring irrelevant stimuli.

Adapted from Miskowiak et al. 2022 and Jespersen et al. 2025. For more details, including visualization of the CAVIR, see Jespersen et al. 2025.

*Screen for Cognitive Impairment in Psychiatry (SCIP)*

SCIP subtask 1, Verbal Learning Test - Immediate (VLT-I) consists of three trials of a ten-word list that participants must remember. SCIP subtask 2, Working Memory Test (WMT) consists of eight recall trials of three consonants. These are distributed among four conditions: no delay, or delays of 3,9 or 18-seconds. Participants must count aloud backwards during the delay time while remembering the three consonants. In SCIP subtask 3, Verbal Fluency Test (VFT), participants are offered 30 seconds to generate as many words as possible that begin with the letter (number and proper nouns are not allowed). In SCIP subtask 4, Verbal Learning Test – Delayed (VLT-D), participants are asked to recall the verbal list from VLT-I after administration of the WMT and VFT. SCIP subtask 5, Psychomotor Speed Test (PST) is a coding task. Six letters with corresponding Morse code dots and dashes are presented in a response key. Participants must complete blank boxes with the code that corresponds to the correct letter. From Purdon (2005) [3].

*Traditional neuropsychological test battery and domains*

**Table ST1**. Overview of the traditional neuropsychological tests assessing processing speed, attention, verbal learning and memory, working memory and executive functions. From Jespersen et al. 2025.

| **Cognitive domains** | **Neuropsychological tests** |
| --- | --- |
| Processing speed | RBANS coding test  Trail Making A |
| Verbal learning and memory | RAVLT subtests (IV total, Immediate recall, delayed recall) |
| Working memory | WAIS Letter-number sequencing  Spatial working memory error (CANTAB)  Spatial working memory strategy (CANTAB) |
| Executive function | Trail Making B One-touch stockings of Cambridge ‘mean choices to correct’(CANTAB)  Fluency test (S and D)  Wisconsin Card Sorting Task ‘perseverative errors’ |
| Attention | Rapid visual processing ‘accuracy’ and ‘mean latency’(CANTAB) RBANS digit span |

*Supplementary methods: statistics*

For tests with lower scores indicating better performance, the scores were inversed to ensure that all scales had the same direction. Measures were inversed for the following tests:

- CAVIR task 4: number of drawers opened until all cutlery and flatware have been found
- Trail Making Test A and B: latency
- Rapid Visual Processing (RVP): latency
- Spatial Working Memory (SWM): Between error and Strategy
- One Touch Stockings of Cambridge (OTS): Mean choice to correct
- Wisconsin Card Sorting Task (WCST): perseverative errors

| **Table S1.** Group comparisons between the patients in the clinical screening sample and replication sample | | |  |
| --- | --- | --- | --- |
|  | **Clinical screening sample** | **Replication sample** |  |
|  | Patients (*n*=68) | Patients (*n* = 70) | *p*-value |
| Sex (female/male) (%) | 62/38 | 50/20 | .229 |
| Age in years, mean (SD) | 31.9 (10) | 33.4 (10.9) | .410 |
| Educational years, mean (SD) | 14.6 (2.5) | 14.4 (2.5) | .709 |
| HDRS-17, mean (SD) | 8.6 (6.7) | 4.4 (3.8) | <.001** |
| YMRS, mean (SD) | 3.1 (3.5) | 1.0 (1.8) | <.001** |
| CAVIR total, mean (SD) | 63.8 (14.4) | 63.8 (16.9) | .997 |
| CAVIR verbal learning and memory, mean (SD) | 12.1 (2.5) | 12.0 (2.2) | .856 |
| CAVIR executive functioning, mean (SD) | 5.7 (3) | 6.1 (2.6) | .502 |
| CAVIR processing speed, mean (SD) | 34.3 (7.1) | 34.01 (9.0) | .798 |
| CAVIR working memory, mean (SD)^a^ | 8.3 (3.4) | 8.8 (5.3) | .517 |
| CAVIR sustained attention, mean (SD) | 20 (6.8) | 20.6 (6.0) | .590 |
| FAST total, mean (SD) | 19 (12.2) | 23.5 (11.3) | .027* |
| **Notes and abbreviations**: The table only includes measures that were collected across both samples and thus allowed for group comparisons. HDRS-17=Hamilton Depression Rating Scale 17-items version. YMRS=Young Mania Rating Scale. CAVIR = Cognition Assessment in Virtual Reality. SCIP=Screen for Cognitive Impairment in Psychiatry. FAST=Functioning Assessment Short Test. M=mean. SD=standard deviation. * = p < 0.05 (two-tailed), ** = p < 0.001 (two-tailed). ^a^For the CAVIR working memory task, lower score indicate better performance | | | |

*Supplementary results*

**References**

1. Miskowiak, K.W., et al., *Cognition Assessment in Virtual Reality: Validity and feasibility of a novel virtual reality test for real-life cognitive functions in mood disorders and psychosis spectrum disorders.* Journal of Psychiatric Research, 2022. **145**: p. 182-189.

2. Jespersen, A.E., et al., *Cognition Assessment in Virtual Reality (CAVIR): Associations with neuropsychological performance and activities of daily living in patients with mood or psychosis spectrum disorders.* Journal of Affective Disorders, 2025. **369**: p. 1053-1063.

3. Purdon, S., *The Screen for Cognitive Impairment in Psychiatry: administration and Psychometric Properties.* Edmonton, Alberta, Canada: PNL Inc, 2005.
